# Equivalent‐quality unflattened photon beam modeling, planning, and delivery

**DOI:** 10.1120/jacmp.v14i4.4211

**Published:** 2013-07-08

**Authors:** Yunfei Huang, Ryan T. Flynn, R. Alfredo, C. Siochi, John E. Bayouth

**Affiliations:** ^1^ Department of Radiation Oncology Division of Medical Physics, University of Iowa Hospitals and Clinics Iowa City IA USA

**Keywords:** flattening filter free, unflattened photon beam, FFF beam modeling, FFF beam planning

## Abstract

The clinical application of the flattening filter‐free photon beam (FFF) has enjoyed greater use due to its advantage of reduced treatment time because of the increased dose rate. Its unique beam characteristics, along with the very high‐dose rate, require a thorough knowledge of the capability and accuracy in FFF beam modeling, planning, and delivery. This work verifies the feasibility of modeling an equivalent quality unflattened photon beam (eqUF), and the dosimetric accuracy in eqUF beam planning and delivery. An eqUF beam with a beam quality equivalent to a conventional 6 MV photon beam with the filter in place (WF) was modeled for the Pinnacle^3^ TPS and the beam model quality was evaluated by gamma index test. Results showed that the eqUF beam modeling was similar to that of the WF beam, as the overall passing rate of the 2%/2mm gamma index test was 99.5% in the eqUF beam model and 96% in the WF beam model. Hypofractionated IMRT plans were then generated with the same constraints using both WF and eqUF beams, and the similarity was evaluated by DVH comparison and generalized 3D gamma index test. The WF and eqUF plans showed no clinically significant differences in DVH comparison and, on average >98% voxels passed the 3%/3mm 3D gamma index test. Dosimetric accuracy in gated phantom delivery was verified by ion chamber and film measurements. All ion chamber measurements at the isocenter were within 1% of calculated values and film measurements passed the 3mm/3% gamma index test with an overall passing rate >95% in the high‐dose and low‐gradient region in both WF and eqUF cases. Treatment plan quality assurance (QA), using either measurement‐based or independent calculation‐based methods of ten clinically treated eqUF IMRT plans were analyzed. In both methods, the point dose differences were all within 2% difference. In the relative 2D dose distribution comparison, >95% points were within 3% dose difference or 3 mm DTA.

PACS number: 87.55.kh

## I. INTRODUCTION

Modern radiation therapy techniques, such as intensity‐modulated radiation therapy (IMRT), hypofractionated stereotactic body radiosurgery (SBRT), and gated treatment, prolong treatment time. Recently, interest has grown in the clinical application of the unflattened photon beam (UF) due to the advantage of reduction in treatment time caused by the increased dose rate. UF beam is also called the flattening filter‐free (FFF) photon beam because the beam is generated by removal of the flattening filter.^1^, ^2^, ^3^, ^4^, ^5^, ^6^ One of the implementation challenges of the UF photon beam is that its characteristics differ significantly from that of the conventional flat photon beam with the flattening filter in place (WF), as documented in previous studies, including one by the author.^7^, ^8^, ^9^, ^10^, ^11^, ^12^, ^13^, ^14^, ^15^, ^16^, ^17^

Some groups have reported UF beam modeling and planning results using various dose calculation algorithms.^3^, ^6^, ^18^, ^19^, ^20^, ^21^, ^22^ Hrbacek et al.^(22)^ described in detail the UF beam modeling process for a TPS using the anisotropic analytical algorithm, and Stathakis et al.^(23)^ commissioned an UF beam for the TPS using the superposition/convolution algorithm. Previous work showed that the integral dose to normal tissue increased in UF beam planning due to the softer energy spectrum.^(5)^ In our work, the energy of the UF beam was tuned to match the original flat beam (eqUF) to avoid the increase in skin dose, as well as to obtain greater increase in dose rate. To our knowledge, there is no published data relating to the accuracy of beam modeling and planning for an eqUF. In addition, existing data on comparison between UF and WF beam planning used mainly the dose volume histogram (DVH) comparison or representative point dose and 2D dose distribution. In this work, we provided a comprehensive 3D dose distribution comparison to quantitatively evaluate the similarity between the WF and eqUF plans.

Furthermore, the stability of the UF beam profile delivered at a high‐dose rate during the initial run‐up time could differ from the WF beam. The increase in dose rate is even greater (four‐ to five‐fold increase) for energy modified eqUF beam^16^, ^24^ compared to a two‐fold increase in the energy unaltered mode. This requires the verification of the UF beam delivery accuracy, especially for gated treatments where the beams are turned on and off frequently. The existing data reported the planning and static delivery accuracy for UF beam IMRT plans for various sites, such as the prostate^(23)^ and head and neck.^(5)^ But sites affected by respiratory motion (such as liver and lung cancer) that need gated technique have yet to be investigated.

Finally, most of the existing data on FFF beams are phantom studies, and we believe that providing clinical data is an essential contribution to the FFF beam radiotherapy.

Previous work by the authors presented a method to obtain an equivalent quality UF photon beam and some dosimetric properties were described. This work focused on the modeling, planning, and delivery of an energy increased eqUF beam. An unflattened photon beam with a beam quality equivalent to a conventional 6 MV photon beam was obtained by removing the flattening filter and tuning the nominal electron energy.^(16)^ The dose rate of the eqUF beam was adjusted to 1500 MU/min. The eqUF beam was commissioned for the commercial Pinnacle^3^ TPS (Philips Medical Systems, Cleveland, OH). Beam model accuracy was evaluated by gamma index test and validated by ion chamber and film measurement. Hypofractionated gated IMRT plans were then generated with the same optimization constraints using the WF and eqUF beams for liver cancer patients. The equivalence between WF and eqUF beam planning was evaluated by the commonly used DVH comparison, as well as a generalized quantitative 3D gamma index comparison. The delivery accuracy in gated treatment at high‐dose rates using the eqUF beam was verified by ion chamber and film measurements in phantom delivery. Finally, data on clinical patient‐specific eqUF IMRT treatment plan quality assurance (QA), using either measurement‐based or independent calculation‐based methods, were analyzed and reported.

## II. MATERIALS AND METHODS

### A. eqUF beam modeling

An eqUF beam with a beam quality matching the conventional 6 MV photon beam was obtained by removing the flattening filter from a Siemens ONCOR Avant‐Garde linear accelerator (Siemens Medical Solutions, Concord, CA) and tuning the nominal electron energy. The dose rate was increased from 300 MU/min with the WF beam to 1500 MU/min with the eqUF beam. Various percent depth dose (%dd) of field sizes ranging from $2\times 2\;{\text{cm}}^{2}$ to $40\times 40\;{\text{cm}}^{2}$ and the corresponding cross‐axis beam profiles at various depths ranging from $d_{\text{max}}$ to 30 cm were collected with a 0.125 cc ionization chamber (PTW Semiflex Type 31010) in a PTW MP3 water tank (PTW Freiburg, Germany). For profiles of field sizes less than $5\times 5\;{\text{cm}}^{2}$, a PTW 0.015 cc pinpoint ionization chamber was also used to avoid the volume‐averaging effect.

The collected beam data were then used for beam modeling on the Pinnacle^3^ TPS. The modeling process followed the method for modeling flattened photon beams presented by Starkschall et al.^(25)^ with a single model used for all field sizes. The physical beam characteristics were used to guide the modeling process. For instance the %dd curves were mainly determined by the penetration ability of the photon beam, which was adjusted by the energy spectrum in the TPS. Since the UF beam was slightly softer than the WF beam, the weight of the low‐energy spectrum could be slightly greater in the eqUF beam model than in the WF beam model. To date no energy spectrum data have been published specifically for the eqUF 6 MV photon beam, so the same energy spectrum was used to model both the WF and eqUF beams. Similarly, large lateral dose falloff was observed, indicating that faster falloff of relative photon fluence away from the central axis (CAX) need to be applied to the eqUF beam model. The reduced head scatter indicates a smaller Gaussian height value, which models the amount of scatter dose contribution. The reduced electron contamination also helps in determining the parameters modeling the electron dose contribution to the buildup region. The final beam modeling parameters were determined by fitting the TPS calculated data to the measured dataset, the same as those used in the WF beam modeling.

**Figure 1:**
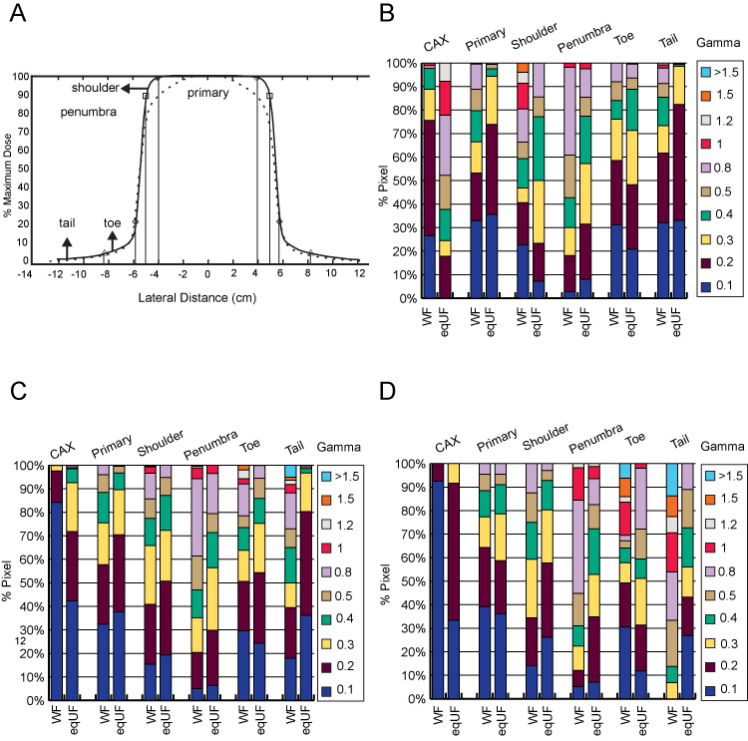
Comparison of 2 mm/2% gamma indices of the WF and eqUF beam model: (a) definition of lateral regions; (b) buildup region; (c) depth 1.5 to 25 cm; (d) deep depth of greater than 25 cm.

In order to verify the quality of the eqUF model, the agreement between the TPS calculated data and the measured data were evaluated by percentage difference and gamma index tests^(26)^ in different lateral regions at different depths: buildup region (≤1.5cm), from 1.5 cm to 25 cm, and >25cm. Laterally, the primary, penumbra, and the out‐of‐field region (tail) were identified using the method of Huang et al.^(16)^ The area between the primary and penumbra region was named as the shoulder, and the area between the penumbra and the tail was named the toe, as shown in Fig. 1(a). An open field and an irregularly blocked field were then created and delivered with the eqUF beam at 1500 MU/min to a solid water phantom. The agreement between the calculated, ion chamber, and film measurement data was evaluated to verify the eqUF beam model.

### B. eqUF beam planning

Ten hypofractionated IMRT liver cancer treatment plans with prescribed doses ranging from 7 Gy to 15 Gy per fraction were created using the WF beam with direct machine parameter optimization (DMPO). For each WF plan, a corresponding plan using the eqUF beam was created using the same beam orientations and optimization constraints/objectives. The numbers of total control points were confined to a 5% difference between the WF and the corresponding eqUF plans. The collapsed cone convolution/superposition (CCCS) algorithm was used as the dose calculation engine.

The equivalence between the generated WF and eqUF plans were evaluated in terms of: 1) the differences in total number of monitor units (MUs); 2) similarity in the open density matrix (ODM) and the dose‐volume histogram (DVH); 3) level of hot or cold spots defined as the percent difference between the maximum dose and the prescribed dose; 4) and the generalized 3D gamma index. The 3D gamma index was calculated by using the 3D dose distribution of the WF plan as the reference dataset and that of the eqUF plan as the evaluation dataset. The 3D gamma index calculation process followed the method presented by Wendling et al.,^(27)^ and the passing rates were calculated with various testing criteria.

### C. Accuracy in gated eqUF beam delivery

The ten hypofractionated IMRT liver cancer treatment plans created with the WF and eqUF beam models were delivered to a solid water phantom with corresponding WF and eqUF beams at dose rates of 300 MU/min and 1500 MU/min, respectively. Gating technique was used with the respiratory trace data retrieved from an archive of previously treated liver cancer patients. The absolute dose delivered to the isocenter in a single fraction was measured by ion chamber and compared to the planned value. The relative 2D dose distribution was also measured with film (KODAK EDR2; Eastman Kodak Company, Rochester, NY) and compared to the planned data set by 2D gamma index tests. The prescribed dose was scaled down in film measurements to be within the dynamic range of the film dosimetry system.

### D. Clinical eqUF patient treatment plan QA

Clinically, both measurement‐based and independent calculation‐based methods are used for patient‐specific IMRT treatment plan QA. For the measurement‐based method, the absolute point dose was measured by ion chamber and the relative 2D dose distribution was measured by film. The measured data were then compared to the planned data, using a similar method to the phantom study. In the independent calculation‐based method, point dose and relative 2D dose distributions were calculated using our in‐house software, and again compared to the planned dataset. In this study, patient‐specific treatment plan QAs for clinically delivered hypofractionated eqUF beam IMRT plans for liver cancer patients were reviewed, with ten cases in each method. The QA results between the two methods were compared, and the clinical applicability of these methods was suggested.

## III. RESULTS

### A. eqUF beam modeling

**Figure 2:**
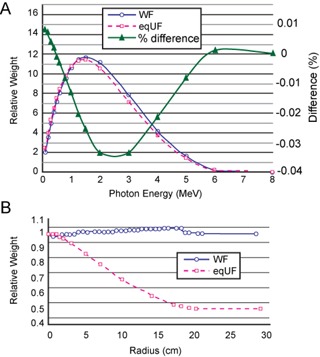
Comparison of the relative energy spectrum (a) and relative incident photon fluence (b) between the WF and eqUF beam model.

Example comparisons of modeling parameters between the WF and eqUF beam models are shown in Fig. 2. The overall distributions of the relative weights of the photon beam energy spectrum (Fig. 2(a)) are similar, with slightly increased weights at the low energy spectrum end (<0.5MeV) and decreased weights for energy spectrum >5MeV in the eqUF beam model. The relative weights of photon incident fluence differ greatly in the eqUF beam model from that of the WF beam model, as shown in Fig. 2(b). While the photon fluence in the WF model was roughly constant across the lateral regions, it is the greatest at the CAX and decreases rapidly away from the CAX in the eqUF beam model. This is expected since the bremsstrahlung radiation is mostly forward peaked in the megavoltage range and the flattening filter attenuates a greater fraction of photons at lateral distances closer to the CAX than further away from it. This effect also results in a difference in the off‐axis softening factor, which is much smaller in the eqUF beam model (0.9 in the eqUF model vs. 9.8 in the WF model), a fact observed by other groups, as well.^(28)^ Differences in other modeling parameters were also observed, such as a 43% lower electron contamination dose at the surface and a 60% smaller Gaussian height in the eqUF beam model.

**Figure 3:**
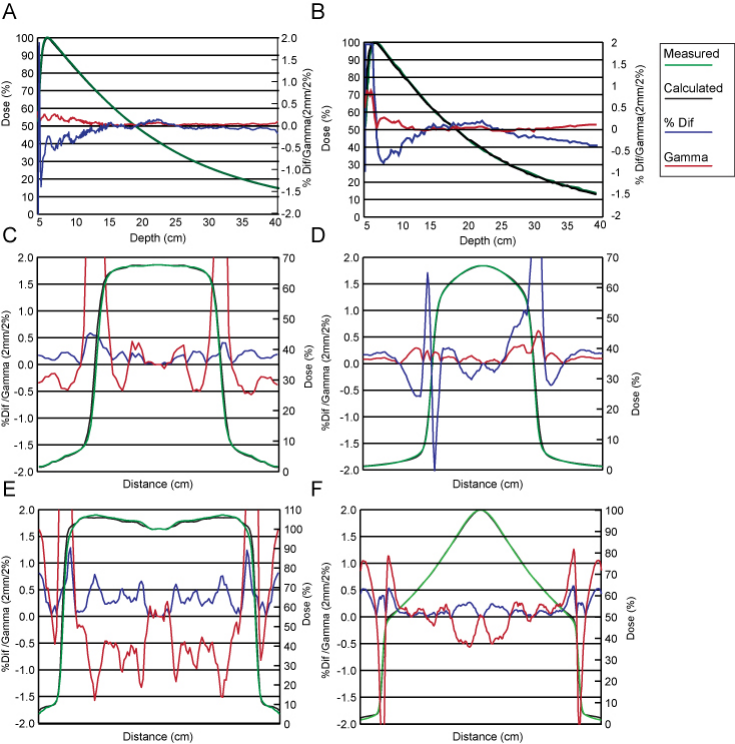
Comparison of calculated and measured data in WF and eqUF beam model. Data fitting of %dd of a 10×10 field in WF beam model (a) and in eqUF beam model (b); data fitting of cross‐axis profile of a 10×10 filed at depth of 10 cm in WF beam model (c) and in eqUF beam model (d); and data fitting of the cross‐axis profile of a 40×40 filed at depth of 1.5 cm in the WF beam model (e) and in the eqUF beam model (f).

Example results of %dd and profile data fitting are shown in Fig. 3. It is evident from Figs. 3(a) and 3(b) that the %dd curves were modeled well within 0.5% in both WF and eqUF beam models, except for the buildup region, where a high gradient exists. Despite the high gradient, the 2mm/2% gamma index tests all passed, including those in the buildup region. Data fitting for the cross‐axis profile of a $10\times 10\;{\text{cm}}^{2}$ field at a depth of 10 cm are comparable between the WF and eqUF beam models, as shown in Figs. 3(c) and 3(d). Differences of <1% were observed for most points except for the high‐gradient region, where the gamma index was smaller than 0.5, indicating a 1 mm distance to agreement (DTA) or 1% dose difference. However, in the case of a larger profile of a $40\times 40\;{\text{cm}}^{2}$ field at a depth of 1.5 cm, the eqUF beam profile was modeled better than the corresponding WF profile, as can be seen from Figs. 3(e) and 3(f).

Detailed data fitting accuracy evaluated by the percentage of points from all %dd and cross‐axis profile data passing the 2mm/2% gamma index test is shown in Fig. 1. Note that the lateral region labeled as CAX represents the %dd data. It is evident that overall the profile data fit better in the eqUF beam model, but the %dd data fit better in WF beam model. In both WF and eqUF models, the region from beyond $d_{\text{max}}$ to 25 cm (Fig. 1(c)), which is the most clinically relevant region, was overall better modeled than in the buildup region (Fig. 1(b)) and deep depth region (Fig. 1(d)). Laterally, the primary region of the profile data fit better than it did for the rest of the regions.

Table 1 summarizes the percentage of points passing the 2mm/2% gamma index test at the CAX and all five lateral regions at three different depth regions. The parentheses show the criteria for the gamma index test (Xmm/X%) through which all points pass. Values in bold indicate an equivalent or superior modeling quality in eqUF beam over the WF beam in the corresponding regions. Again, we see that the WF beam was better modeled for %dds, while the eqUF beam was better modeled for cross‐axis profiles. The maximum criteria of gamma index tests for all points to pass is 2.4 mm/2.4% for the eqUF beam model and 5 mm/5% for the WF beam model. In the most clinically relevant depth, the depth beyond $d_{\text{max}}$ and less than 25 cm, all the %dd data (CAX) and the primary profile data pass the 2mm/2% gamma index test in both the WF and UF beam models. Overall, the eqUF beam was modeled slightly better, as the average passing rate of the 2%/2mm gamma index test was 96% in the WF beam model and 99.5% in the eqUF beam model.

**Figure 4:**
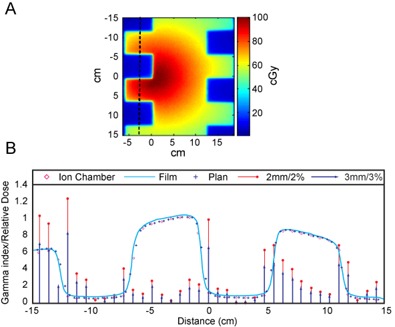
2D dose distribution (a) of the film measurement of an irregular field exposed to the eqUF beam, and comparison (b) of the profiles passing the middle of the blocked region (as indicated by the dashed line in (a)) of the irregular field: film measurement (solid line); ion chamber array measurement (diamond); Pinnacle^3^ calculation (star); 3mm/3% (triangle) and 2mm/2% (circle) gamma index tests between ion chamber measurement and TPS calculation.

Figure 4 shows the relative 2D dose distribution of the irregular field measured by film (Fig. 4(a)) and the profile passing the middle of the blocked regions (the dashed line in Fig. 4(a)) obtained by TPS calculation, ion chamber measurement, and film measurement, normalized to the maximum dose of the ion chamber measurement (Fig. 4(b)). The ion chamber measurements agreed better on average with the Pinnacle^3^ calculated value than the film measurement did. Also seen in Fig. 4(b) are the 3mm/3% and 2mm/2% gamma indices of the ion chamber measurement compared to the Pinnacle^3^ calculation at each measurement point. With the exception of one point in the high‐dose gradient region, which passed the 3mm/3% gamma index test, all other points including regions both inside and outside the field pass the 2mm/2% gamma index test.

**Table 1:** Comparison of the percentage of data points passing the 2mm/2% gamma index test. The criteria of gamma index test at which all points pass are show in the parenthesis. Numbers in bold represent equally well or better results in eqUF model

*Pass Rate*	Depth≤1.5cm		Depth1.5cm–25cm		Depth>25cm	
2mm/2%	WF(mm/%)	eqUF(mm/%)	WF(mm/%)	eqUF(mm/%)	WF(mm/%)	eqUF(mm/%)
CAX	100(2)	92.2(2.4)	100(0.8)	100(2)	100(0.4)	100(0.6)
Primary	100(2)	100(1.6)	100(1.6)	100(1.6)	100(1.6)	100(1.6)
Shoulder	91.4(3)	100(1.6)	99.3(3)	100(1.6)	100(1.6)	100(1.6)
Penumbra	100(2)	100(2)	98.9(3)	100(2)	98.3(2.4)	98.8(2.4)
Toe	100(2)	100(2)	94.2(3)	100(2)	83.6(3.4)	100(2)
Tail	99.3(4)	100(1.6)	91.9(5)	100(1.6)	70.6(4.4)	100(1.6)

### B. eqUF beam planning

**Figure 5:**
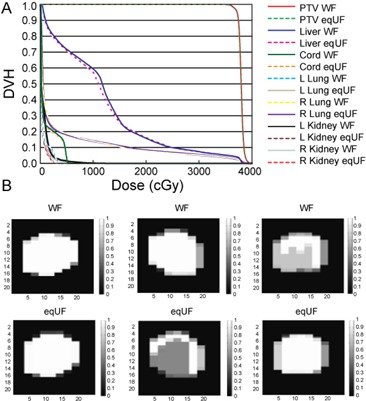
Example DVH comparison (a) between the WF and eqUF plans; comparison (b) of ODM for given beams (top row: WF cases; bottom row: eqUF cases). They could be very similar in WF and eqUF cases (left) or very different (middle and right).

DVH comparison between the WF and eqUF plans for the ten IMRT liver cancer cases showed no clinically significant differences, as seen from the example in Fig. 5(a). This indicated that clinically equivalent plans could be generated using WF or eqUF beams with the same beam orientation and optimization objectives. Figure 5(b) shows an example of ODM comparisons for the same beams in the WF and eqUF plans. Very similar (left column) or very different (middle and right column) ODMs were all possible, indicating that the TPS is able to modulate the intensity equally well for WF and UF beams.

Hot spots evaluated by the average percentage differences between the maximum dose and the prescribed dose were similar for the ten WF and ten eqUF plans: 7.1%(STD=2.4%) higher in WF plans and 7.2%(STD=1.6%) higher in eqUF plans. The number of total MUs in eqUF plans differed from those in the WF plans by −6.3% to 27.2%, with an average of 7.4%(STD=9.1%).

**Figure 6:**
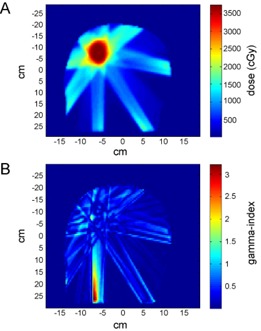
An example of (a) 2D dose distribution of an eqUF beam IMRT plan and (b) the 3D gamma index map comparing the WF the eqUF plans at the same plane.

The equivalence in WF and eqUF beam planning was further verified quantitatively using a 3D gamma index test. An example 3D gamma index map of an x‐y plane is plotted in Fig. 6(b), along with the 2D dose distribution in that plane (Fig. 6(a)). A small portion of voxels failed the 3mm/3% gamma index test indicating a 3% or larger dose difference or 3 mm or larger DTA at the comparison point between the WF and eqUF plans. However, Fig. 6(a) shows that voxels with relatively large differences (large gamma values) were distributed mainly in the low‐dose region out of the PTV. Taking the whole calculation volume of this plan into account, 99.7% voxels passed the 3mm/3% test and 92.4% voxels had gamma indices 0.3 or smaller, indicating <1% dose differences or <1mm DTA. For the ten cases compared in this study, the average passing rate in the 3mm/3% 3D gamma index test was about 98%, with 95% voxels having gamma indices 0.5 or smaller. This verified that the 3D dose distributions were very similar between plans generated with WF and eqUF beams.

### C. Accuracy in gated eqUF beam delivery

**Figure 7:**
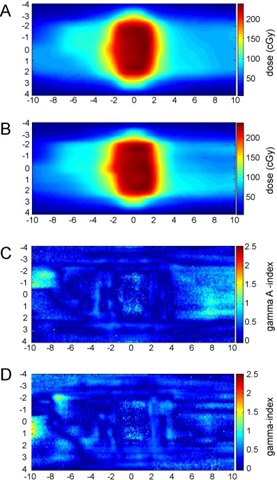
Example calculated 2D dose distribution of a WF plan (a) and of an eqUF plan (b); and 3mm/3% 2D gamma index map comparing the film measurement to the plan in the WF case (c) and in the eqUF case (d).

The agreement between the TPS planned dose and the gated delivered dose was validated by ion chamber and film measurements in phantom delivery. The difference between the prescribed dose and the actual delivered dose to the isocenter as measured by ion chamber was within 3% in all ten WF and ten eqUF cases. On average, the delivered dose was 0.8%(STD=1.3%) higher than the prescribed dose in WF cases and 0.8%(STD=1.1%) lower in eqUF cases. The relative 2D dose distribution in film measurements was compared to the calculated dataset by 3mm/3% gamma index test. Figure 7 shows examples of the calculated 2D dose distribution and the gamma index map for a WF case and a corresponding eqUF case. Even though the gamma index maps differ slightly between the WF and eqUF cases, both passed the 3mm/3% gamma index test with only a small portion in the low‐dose region out of the tumor volume failing the test. The average 3%/3mm and 5%/5mm gamma ‐index passing rate is 98.3%(STD=2.4%) and 99.7%(STD=0.9%), respectively, for the ten WF plan delivery. The corresponding passing rate for the eqUF plan delivery was 98.6%(STD=1.2%) and 99.9%(STD=0.03%), respectively. The difference between the WF and eqUF cases are insignificant at both levels of 3%/3mm(p=0.6) and 5%/5mm(p=0.3). This confirms that the eqUF beam plan can be delivered at a high‐dose rate in gated mode with a similar dosimetric accuracy as that in the WF deliveries.

### D. Clinical eqUF patient treatment plan QA

**Figure 8:**
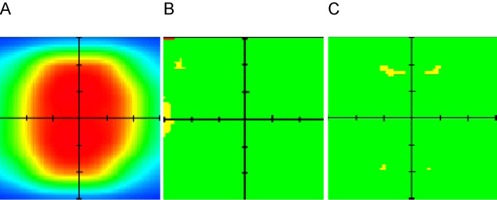
An example of clinical patient‐specific eqUF beam IMRT treatment QA: (a) TPS‐calculated 2D dose distribution; (b) map of 3% dose difference and 3 mm DTA comparison between TPS and film measurements and (c) between TPS and independent calculation.

Review of the clinically treated eqUF beam IMRT plans showed strong agreement in both measurement‐based method (between planned and measured dataset) and the independent calculation‐based method (between planned and independently calculated data). In the ten measurement‐based QAs, the average ratio of point dose measured by ion chamber at the isocenter to the planned dose was 1.004(STD=0.018). The agreement between the planned 2D dose distribution and the relative film measurements were within 3% dose difference or 3 mm DTA for 95% of all the pixels, excluding the low‐dose region. Similarly, the average ratio of the independently calculated point dose to the planned dose was 1.006(STD=0.012). The percentage of pixels with agreement between the planned 2D dose distribution and the independent calculation being within 3% dose difference or 3 mm DTA was also 95% or greater. Figure 8 shows an example of the planned 2D dose distribution (Fig. 8(a)) and the agreement between the TPS and the film measurement (Fig. 8(b)) or independent calculation (Fig. 8(c)). It is evident that the relative 2D dose distribution in both film measurement and independent calculation agreed well with the TPS calculation, as pixels in the high‐dose low‐gradient region (indicated in red in Fig. 8(a)) were mostly within 3% dose difference and 3 mm DTA (indicated in green in Figs. 8(b) and 8(c)). The yellow regions in Figs. 8(b) and 8(c) indicate only one of the criteria was met and that either the dose difference or the DTA was out of tolerance. Compared to Fig. 8(a), it is apparent that these regions are located mainly in the low‐dose or high‐gradient region and clinically less critical. This validated that the performance of Pinnacle^3^ in dose calculation for the eqUF beams meets the current state‐of‐the‐art clinical requirements. It also indicated that the measurement‐based and independent calculation‐based patient specific IMRT QA work equally well for the eqUF beam IMRT treatment plans.

## IV. DISCUSSION

In the eqUF beam model, the average 2mm/2% gamma values of cross‐axis profile data fitting were smaller than that of the WF model. This is expected since the absence of the flattening filter can facilitate the UF beam modeling.^5^, ^28^ All scatter‐related modeling processes become less complex with the flattening filter removed, as there is less lateral beam softening, less variation in head scatter across different field sizes, and less variation in beam profiles across the different depths.^(16)^ One apparent disadvantage is that the gamma values of %dd data fitting, though still small, were increased in the eqUF beam model. This is likely the result of the use of the same photon energy spectrum as the WF beam or inadequate adjustment of the relative weights of the energy spectrum. The %dd data fitting could be improved further if the energy spectrum specific to the eqUF beam was used, or the energy spectrum distribution was adjusted more aggressively based on the data fitting instead of attempting to maintain the similarity in energy spectrum distribution of the WF beam model. It is also worth pointing out that the beam model showed improved results in the tail section for the eqUF beam, as seen in Fig. 1. This is probably due to the scattered radiation depositing dose outside the field, which is difficult for the planning algorithm to accurately model, and is reduced when the flattening filter is removed. Increased accuracy of dose modeling in the tail section provides an advantage in IMRT planning and delivery, where the out‐of‐field dose is of concern.

During this study both MU increase and decrease occurred, even though the overall total number of MUs increased slightly with the use of the eqUF beam. This appears to conflict with the results reported by Stathakis et al.,^(23)^ where a significant reduction in total MU was observed. This discrepancy may be explained as being due to the difference in linac output calibration used in each study: 2 cGy/MU (or more) was used for the unflattened beam in the study by Stathakis et al., but only 1 cGy/MU was used for the WF beam in our study.

Our clinical patient‐specific eqUF IMRT treatment plan QA showed equivalence between the measurement‐based and independent calculation‐based methods. This indicates that either method is valid. Thus individual institutions can prepare a patient treatment plan QA program for unflattened beam IMRT plans compatible with existing programs to meet their specific requirements.

## V. CONCLUSIONS

The energy increased equivalent quality unflattened photon beam can be modeled equally well for Pinnacle^3^ TPS as for the flat beams. Our current TPS generated eqUF IMRT plans that are clinically no different from the WF plan. Dosimetric accuracy in gated IMRT phantom treatment was also similar when delivered at 300 MU/min using WF beam and at 1500 MU/min using the eqUF beam. The clinical eqUF IMRT patient treatment plan QA results showed strong agreement between TPS plans and measurements or independent calculations, with results being equivalent using either method.
